# Effectiveness of Approaches to Increase Physical Activity Behavior to Prevent Chronic Disease in Adults: A Brief Commentary

**DOI:** 10.3390/jcm8030295

**Published:** 2019-03-01

**Authors:** Juliano Schwartz, Ryan Rhodes, Shannon S. D. Bredin, Paul Oh, Darren E. R. Warburton

**Affiliations:** 1 Physical Activity Promotion and Chronic Disease Prevention Unit, University of British Columbia, Vancouver, BC V6T 1Z4, Canada; juliano@alumni.ubc.ca (J.S.); shannon.bredin@ubc.ca (S.S.D.B.); 2 CAPES Foundation, Ministry of Education of Brazil, Caixa Postal 250, Brasília–DF 70040-020, Brazil; 3 School of Exercise Science, Physical and Health Education, University of Victoria, Victoria, BC V8P 5C2, Canada; rhodes@uvic.ca; 4 Cardiac Rehabilitation and Prevention Program, Toronto Rehabilitation Institute, University Health Network, Toronto, ON M4G 1R7, Canada; paul.oh@uhn.ca

**Keywords:** physical inactivity, behavior change, risk factors

## Abstract

Effective behavior change approaches are necessary to help individuals avoid or mitigate risk factors, engage in behavioral patterns that lead to better health, and consequently, prevent several chronic diseases. Physical inactivity is considered one of the most harmful risk factors for chronic medical conditions, and although different strategies are used to tackle this behavior, not all of them lead to the expected or desired results. This brief commentary examines recent approaches aimed at physical activity behavior change. We find that a combination of strategies focusing on streamlining the access to physical activity seems to be more effective than individual approaches, in order to increase physical activity engagement to prevent chronic diseases in adults.

## 1. Introduction

There has been a concerted effort in public health messaging about the health benefits of physical activity [1,2]. As a result, research suggests that there is an increased knowledge base about the importance of physical activity in relation to health promotion and chronic disease prevention across end-users and key stakeholders [3,4,5]. Despite this increased general awareness, levels of physical activity have not increased markedly globally, while sedentary behavior has progressively increased [1,6]. 

Physical inactivity is shown to be a preeminent risk factor in terms of morbidity and premature mortality, being responsible for over five million deaths each year around the world [7]. Additionally, data from 2013 showing the global economic burden of physical activity of more than INT $67 billion (in terms of health care cost and losses in productivity) makes the pandemic of physical inactivity a critical contemporary issue [1,8]. As such, there has been an increase in physical activity policies at governing institutions, which meet the World Health Organization’s goals of reducing physical inactivity levels, as well as the associated premature mortality from chronic diseases [9,10,11]. 

Different determinants may lead a person to be physically active or not [12]. Early studies focused on motivation, showing that some adults do not engage in regular physical activity owing to a relative lack of encouragement [13,14,15]. Later, attention was given to environmental factors (e.g., built environment), which showed that addressing factors related only to individuals might not be enough to change physical activity behavior [16,17,18]. Recently, literature has highlighted the importance of affective judgments in physical activity participation, (i.e., how pleasure and enjoyment influence people’s choices regarding becoming more active) [19,20]. This research has demonstrated great promise in the enhancement of physical activity behavior.

It is important to highlight the differences between sedentary behavior and physical inactivity. The terms “sedentary” and “physically inactive” are often used interchangeably and without the full appreciation for how these are distinct constructs, each carrying their own independent risks [2,21]. Sedentary behavior is classically defined as time spent in behaviors in the sitting, lying down, or reclined position (such as sitting, driving a car, and watching television) that require low energy expenditure (i.e., ≤1.5 METs) [2,21]. Whereas, physical activity refers to all leisure and non-leisure time body movements leading to an increased energy expenditure from rest (excluding low-energy expenditure activities done while sitting, reclining, or lying down) [21,22]. Physical activity has four broad domains including occupational (work-related), domestic (house work, yard work, child care, chores), transportation (bicycling or walking), and leisure-time (discretionary or recreational time for physical activity, sports, exercise, and hobbies) [21]. Physical inactivity is often operationally defined as not meeting international recommendations (such as 150 min per week of moderate-to-vigorous intensity physical activity) [5]. However, recent authors have argued against the potential perils of this threshold-based messaging, and indicate that the health benefits could still be acquired for much lower volumes, intensities, and/or durations [2,23]. 

When considering sedentary behaviors, most people (with the exception of those confined to bed rest or dependent on others) will engage in some form of physical activity throughout the day [21,24]. It is also important to acknowledge that a physically active person can also be highly sedentary [25]. Although adults who usually spend too much time in sedentary behaviors (such as screen time and seated office work) are often not physically active, other individuals engage in some moderate-to-vigorous physical activity throughout the week, but spend long periods in sedentary activities in the rest of their time [26,27]. As such, a clear separation of sedentary and physical inactivity definitions is required. 

The health risks of routinely engaging in sedentary behaviors have been increasingly examined. Links to an increased risk for medical conditions such as weight gain, diabetes, heart diseases, and cancer [28,29] and premature mortality are well established [30,31]. Public health messaging related to physical activity promotion has thus been modified to include more inclusive statements such as “Move More, and Sit Less” [2]. However, recent authors have argued that the increased attention to sedentary behaviors has taken away from some of the attention paid to the importance of regular physical activity participation [1]. It is clear that routine physical activity has its own benefits in reducing chronic disease risk, but importantly, it can also decrease the increased risks associated with sedentary behaviors [1,7], and possibly eliminates the elevated risk of mortality related to prolonged sitting times [7]. In fact, according to Ekelund [32], one hour of moderate physical activity a day is suggested to eliminate the increased risk of mortality imposed by eight hours of daily sitting time. This physical activity effect is particularly valuable when it is unavoidable to be sitting daily for extended periods [32].

Although the health benefits of physical activity are clear [5], not all approaches to increase physical activity participation lead to the expected results. Accordingly, the following section outlines various approaches used to increase physical activity participation.

## 2. Building on Behavior Change Approaches to Increase Physical Activity Participation

Different behavior change theories address how a behavior is adopted [33]. While theories explain the mechanisms to achieve change of behavior, they require specific techniques to grant change [12,34]. Dishman [35], and Rhodes and Nigg [36], highlight the fact that physical activity is a complex behavior, different from all others in the health area: e.g., it is an acquisition behavior, whereas, smoking and drug use are extinction habits; and it requires more time and dedication than behaviors such as oral care (flossing and tooth brushing). Yet, the theories and models applied in the physical activity field were usually developed to be used in other areas, which is possibly linked to the fact that there is no consensus about the best strategies to change physical activity behavior [36,37].

Recently, different strategies have been receiving attention to increase physical activity levels in adulthood. These include, among others, the use of technology [38], less traditional physical activity modalities for public health purposes, such as martial arts and combat sports [39], and programs that incorporate different intensities [40]. There are also a number of recent publications that highlight behavioral techniques to promote physical activity [12,41].

### 2.1. Focus on Small Quantities of Physical Activity

International guidelines recommend a threshold of a minimum of 150 minutes of moderate to vigorous physical activity per week to achieve numerous health benefits [5,42]. This message however may impose an avoidable and unnecessary barrier to healthy living for those who could benefit from simply becoming more active at lower levels, since research has shown that such a threshold actually does not seem to exist [2,43]. Even small quantities of physical activity are linked with reduced premature mortality as well as the primary and secondary prevention of cardiovascular disease, diabetes, and cancer, among various other chronic conditions [44,45]. In fact, Hupin et al. [46] revealed that the first 15 minutes of physical activity lead to the greatest benefits to the health of older adults. Furthermore, the clear message around the importance of simply becoming more physically active is of crucial importance for vulnerable groups, such as minorities, women, and those with low socio-economic status [44,47]. Regardless of the amount, the fact is that physical activity levels in the general population must increase, and this can be done not only at leisure time in a structured manner, but also during other moments and in less conventional ways throughout the day, such as during work and even when commuting [48].

### 2.2. Increasing Self-Regulation

Approaches involving self-management (e.g., learning approaches to engaging in positive health behaviors) promote better results in terms of behavior change than passive approaches (e.g., advice about benefits of exercise) [49]. In fact, short and long term physical activity behavior increases when self-regulatory techniques are implemented [50,51]. Among the techniques of self-regulation–a process focused on achievement and maintenance of one’s goal–self-monitoring (record keeping about a specific behavior) is strongly suggested to increase physical activity levels [49,50,52]. An example of how self-monitoring can boost physical activity participation is through the use of wearable technology, such as pedometers and activity trackers that include accelerometry technology [53]. Indeed, the use of the pedometer was shown to develop autonomous motivation for engagement—not only during a physical activity program, but also for the maintenance of physical activity after the intervention [54]. Self-monitoring, when used together with at least one more self-regulatory approach, such as goal setting (elaboration of a meticulous plan about the action to be taken) and behavior feedback (comparing one’s performance with similar others, as well as comparing the current performance with previous ones) form a cluster considered highly effective in increasing physical activity participation [12,49].

### 2.3. Nonconscious Processes

Conscious regulatory processes to engage in physical activity, such as intention (plans) and self-efficacy (beliefs), have been shown to explain just a small proportion of physical activity behavior [55]. At the same time, an increasing number of publications highlight the importance of unconscious processes (unaware mental operations) to increase physical activity participation [55,56,57,58]. Among other nonconscious processes, such as automatic associations and priming effects, more research attention has been given to habits [59,60,61]. Although more research is warranted to clarify the amount of time needed for the formation of a habit (an automatic sequence of actions in response to particular cues), initial studies in physical activity showed that around six weeks might be a reasonable period [62,63]. Key to this process of consistent practice of physical activity is the fact that the workout should be enjoyable for the participant [58,62].

### 2.4. Internet and Telephone

Conn et al. [64] suggest that interventions mediated by the Internet or telephones are less effective than the ones delivered face-to-face. Although Rhodes et al. [12] agree with these findings, they point out that practical issues limiting delivery should also be contemplated, especially when financial and human resources are not available to support in-person counseling. These are important considerations, since the high global burden of chronic diseases in low resource settings demands low-cost interventions targeting as many people as possible [10,65]. Accordingly, approaches using electronic health (eHealth) and mobile health (mHealth), such as mobile devices and smartphone applications (apps) are cost-effective in promoting physical activity engagement [66]. Indeed, well implemented telehealth and web-based tools (including but not being limited to telephone lines and e-mail communications) have been shown to properly translate how the best scientific evidence can be utilized by end users, in an effective and dynamic manner [67,68,69].

### 2.5. Increased Accessibility to Facilities and Environments

Urban planning, such as accessibility to fitness facilities and welcoming community environments is imperative in terms of physical activity engagement [12,70]. Interventions focusing on enhancing the access to fitness facilities have been shown to increase the levels of physical activity in adults of different ages, contributing to the primary and secondary prevention of chronic diseases [70,71]. In that regard, proximity to nearby parks, as well as access to public transportation play an important role [72].

### 2.6. Individual or Collective Approaches

Rhodes et al. [12] point out that interventions focusing only on physical activity are more effective than those targeting multiple behaviors. This is in accordance with findings pointing out that multiple health behavior change approaches still need more scientific support, since individuals possibly do not value the benefits of such approaches enough [73,74]. However, physical inactivity is amongst a few other fundamental health behaviors associated with the development of chronic conditions, such as an unhealthy diet and smoking [3]. Additionally, physical activity alone is not enough to prevent obesity, which is another risk factor for chronic diseases [75,76,77]. In fact, interventions tackling multiple behaviors for a common health goal, such as the prevention of certain chronic conditions, have led to significant change in multiple behaviors [74].

### 2.7. Social Pressures and Passive Approaches

When it comes to strategies considered unsuccessful to change physical activity, Michie et al. [49] agree with Conn et al. [64] by stating that passive strategies (when the participants simply receive information about the importance of being physically active) do not increase physical activity. Also, research suggests that interventions targeting how one perceives pressures from significant others—such as family and media—to perform a behavior (subjective norms) does not lead to a change in physical activity [36,78].

## 3. Conclusions

Overall, recent findings indicate that no isolated strategy is better than another concerning an increase in physical activity engagement [78]. Instead, it seems that a combination of approaches can lead to better outcomes [12,79]. In order to increase physical activity participation aiming at chronic disease prevention, the optimal strategy should make use of a number of proven approaches that are grounded in relevant theories and targeting the individual and their environment.
